# Feedback activation of STAT3 limits the response to PI3K/AKT/mTOR inhibitors in PTEN-deficient cancer cells

**DOI:** 10.1038/s41389-020-00292-w

**Published:** 2021-01-05

**Authors:** Jian Wang, Xiaoye Lv, Xiutian Guo, Yanbo Dong, Peipei Peng, Fang Huang, Peng Wang, Haoqian Zhang, Jianguang Zhou, Youliang Wang, Bo Wei, Zeng-Fu Shang, Shanhu Li

**Affiliations:** 1 Department of Cell Engineering, Beijing Institute of Biotechnology, 100850 Beijing, China; 2 Department of General Surgery, The General Hospital of People’s Liberation Army, 100853 Beijing, China; 3 Department of Spine Surgery, Jinan No. 4 Hospital, 250029 Jinan, China; 4 Shanghai municipal Hospital of Traditional Chinese Medicine, Shanghai University of Traditional Chinese Medicine, 200071 Shanghai, China; 5 Binzhou Medical University, 264003 Yantai, China; 6 State Key Laboratory of Radiation Medicine and Protection, School of Radiation Medicine and Protection, Medical College of Soochow University, Collaborative Innovation Center of Radiation Medicine of Jiangsu Higher Education Institutions, Soochow University, 215123 Suzhou, China

**Keywords:** Cancer therapeutic resistance, Cancer genetics

## Abstract

The PI3K/AKT/mTOR signaling pathway is constitutively active in PTEN-deficient cancer cells, and its targeted inhibition has significant anti-tumor effects. However, the efficacy of targeted therapies is often limited due to drug resistance. The relevant signaling pathways in PTEN-deficient cancer cells treated with the PI3K/mTOR inhibitor BEZ235 were screened using a phosphokinase array, and further validated following treatment with multiple PI3K/AKT/mTOR inhibitors or AKT knockdown. The correlation between PTEN expression levels and STAT3 kinase phosphorylation in the tissue microarrays of gastric cancer patients was analyzed by immunohistochemistry. Cell proliferation and clonogenic assays were performed on the suitably treated PTEN-deficient cancer cells. Cytokine arrays, small molecule inhibition and knockdown assays were performed to identify related factors. PTEN-deficient tumor xenografts were established in nude mice that were treated with PI3K/AKT/mTOR and/or STAT3 inhibitors. PTEN deficiency was positively correlated with low STAT3 activity. PI3K/mTOR inhibitors increased the expression and secretion of macrophage migration inhibitory factor (MIF) and activated the JAK1/STAT3 signaling pathway. Both cancer cells and in vivo tumor xenografts showed that the combined inhibition of PI3K/AKT/mTOR and STAT3 activity enhanced the inhibitory effect of BEZ235 on the proliferation of PTEN-deficient cancer cells. Our findings provide a scientific basis for a novel treatment strategy in cancer patients with PTEN deficiency.

## Introduction

The recent advances in cancer research, and identification of pro-oncogenic factors and signaling pathways have initiated a new era of precision anti-cancer treatment^1,2^. A series of highly specific targeted therapeutic drugs have been developed that have shown encouraging results against recalcitrant tumors in clinical trials. Prominent examples include epidermal growth factor receptor (EGFR) kinase inhibitors (Afatinib, Gefitinib, and Osimertinib) for treating EGFR-mutated non-small cell carcinoma (NSCLC), the abelson murine leukemia viral oncogene homolog (ABL) kinase inhibitor imatinib against chronic myelogenous leukemias harboring the *BCR-ABL* fusion oncogene, and BRAF inhibitors (dabrafenib and vemurafenib) for the treatment of melanoma with V600E or V600K BRAF mutations^1,3,4^. Unfortunately, in most patients, tumors are refractory to targeted therapies (de novo resistance). Even if an initial response to targeted therapies is obtained, the vast majority of tumors subsequently become refractory (acquired resistance) and patients eventually succumb to disease progression. Several potential mechanisms of resistance against targeted therapies have been proposed using cancer cell models. One possibility is that a second mutation event in the targeted oncogene can restore its functions and also render the drug specific for the initial mutation redundant. For example, secondary mutations in EGFR, ABL, and anaplastic lymphoma kinase (ALK) block the interaction between the targeted drugs and the ATP-binding pocket^5^. Furthermore, mutations or amplification of signaling pathway molecules upstream or downstream of the targeted site can also neutralize the effect of targeted drugs. In fact, cancer cells often initiate other compensatory signaling pathways during chemotherapy to reactivate the pathways related to cancer cell growth and proliferation, and eventually develop drug resistance^6,7^.

The PI3K/AKT/mTOR cascade relays signals from several receptor tyrosine kinases (RTKs) following ligand binding, and regulates cell metabolism, proliferation, survival, and migration through downstream effector molecules. It is often constitutively activated in cancer cells through different molecular mechanisms, such as mutations (EGFR) and amplification (human epidermal growth factor receptor 2, HER2) of RTKs, mutations in downstream molecules such as phosphatidylinositol-4, 5-bisphosphate 3-kinase catalytic subunit alpha (PIK3CA) or AKT, or loss of function of the tumor suppressor molecule phosphatase and tensin homolog (PTEN)^8^. Given the oncogenic role of the PI3K/AKT/mTOR signaling pathway, this pathway presents an attractive candidate for targeted therapeutics. In recent years, many small molecule inhibitors targeting PI3K, AKT, and the downstream effector mTOR have been developed; several are in clinical trials, and some have been approved for therapy by FDA, of which idelalisib has been approved for the treatment of relapsed chronic lymphocytic leukemia (CLL), relapsed follicular B-cell non-Hodgkin’s lymphoma (FL), and relapsed small lymphocytic lymphoma (SLL)^9^ and Everolimus is approved in combination with exemestane to treat postmenopausal women with advanced hormone receptor positive, HER2-negative breast cancer^10^. Although small molecule inhibitors of PI3K/AKT/mTOR have been effective in clinical trials, their therapeutic efficacy is still limited by intrinsic and acquired drug resistance of the tumors. Therefore, elucidating the mechanisms underlying resistance to PI3K inhibitor can provide rationale for combination therapies and alternative therapies.

The PTEN gene encodes a phosphatase that inhibits the PI3K/AKT/mTOR signaling pathway by increasing accumulation of phosphatidylinositol-3,4, 5-triphosphate (PIP3)^11,12^. Preclinical studies show that PTEN deficiency sensitizes some cancer subtypes to PI3K pathway inhibitors^13,14^. However, the PTEN-deficient cancer cells mainly rely on the p110β isoform of class IA PI3K and its downstream effectors to transmit signals, which can trigger resistance against the PI3K p110α inhibitors by restoring the PI3K/AKT signaling pathway^15–17^. In addition, even in PTEN-deficient cancer cells, a selective inhibitor of PI3Kβ only led to transient suppression of, followed by a significant rebound in phospho-AKT levels, which were attributed to the upregulation of the IGFR1–IRS1–p110a signaling cascade. Therefore, simultaneous inhibition of p110β and p110α can potentially block the PI3K/AKT signaling pathway and suppress tumor growth^18^. However, there are reports of the unresponsiveness of PTEN-deficient cancer cells to pan-PI3K inhibitors. For example, PTEN-deficient breast cancer cells are resistant to the pan-PI3K inhibitor GDC-0941 and PI3K/mTOR dual inhibitor BEZ-235, unlike those harboring mutations in PIK3CA and HER2^19–21^. Similarly, the PIK3CA-mutated and HER2-mutated breast cancer cells are selectively sensitive to mTOR allosteric and kinase inhibitors, while the PTEN-deficient cells are resistant^22^. A study using PDX models of gastric cancer found that a single dose of the pan-AKT kinase inhibitor AZD5363 inhibited the growth of SGC100 PDX harboring the PI3KCA H1047R activation mutation by 60%, whereas only 23% inhibition was seen in the SGC020 PDX model with PTEN deficiency. This indicated that PI3KCA mutation rather than PTEN deficiency predicts sensitivity of gastric cancer cells to AZD5363^23^. Interestingly, the PI3K and androgen receptor (AR) signaling pathways have an interactive feedback regulation in PTEN-deficient prostate cancer cells, and inhibition of either can activate this loop and thus limit the efficacy of single drug treatment. In fact, combined inhibition of the PI3K/AR signaling pathway has demonstrated better therapeutic effects^24,25^. These studies point to a complex drug resistance mechanism in the PTEN-deficient cancer cells against PI3K/AKT/mTOR inhibitors.

The STAT3 protein plays an important role in cell proliferation, differentiation, survival, inflammatory response, immunity, and angiogenesis, and is aberrantly activated in breast, prostate, head and neck, colon, lung, and multiple myeloma cancer cells. Persistent STAT3 activation promotes cell-cycle progression, tumor invasion, metastasis, and angiogenesis^26–28^. In addition, feedback activation of STAT3 by tyrosine kinase inhibitors (TKIs) often weakens their efficacy, and eventually results in drug resistance^29^. For instance, erlotinib can directly activate STAT3 activity in the EGFR-mutated lung cancer cells and promote drug resistance. Similarly, the TKI-mediated STAT3 activation is the basis of chemoresistance in HER, MET, and ALK tumor models. RTKs relay signals mainly via the PI3K/AKT/mTOR and RAS/MEK/ERK pathways. Inhibition of ERK kinase activity results in STAT3 feedback activation through the fibroblast growth factor receptor (FGFR) and interleukin 6 (IL-6)/JAK1 signaling pathway, while the PI3K/AKT signaling pathway can positively activate STAT3 kinase activity^30^. In some solid tumor cells, feedback activation of STAT3 by HDAC inhibitors induces drug resistance by upregulating leukemia inhibitory factor receptor, which activates the JAK1/STAT3 signaling pathway^31^. While increasing evidence supports the compensatory activation of STAT3 kinase and its downstream signaling pathways is a common cause of resistance of different cancer cell types and oncogenic contexts to targeted therapies, whether it can play role in dampening the response to the PI3K inhibitors in PTEN-deficient cancer cells remains an unanswered question.

## Results

### PTEN deficiency inhibits STAT3 activity in cancer cells

**Fig. 1:**
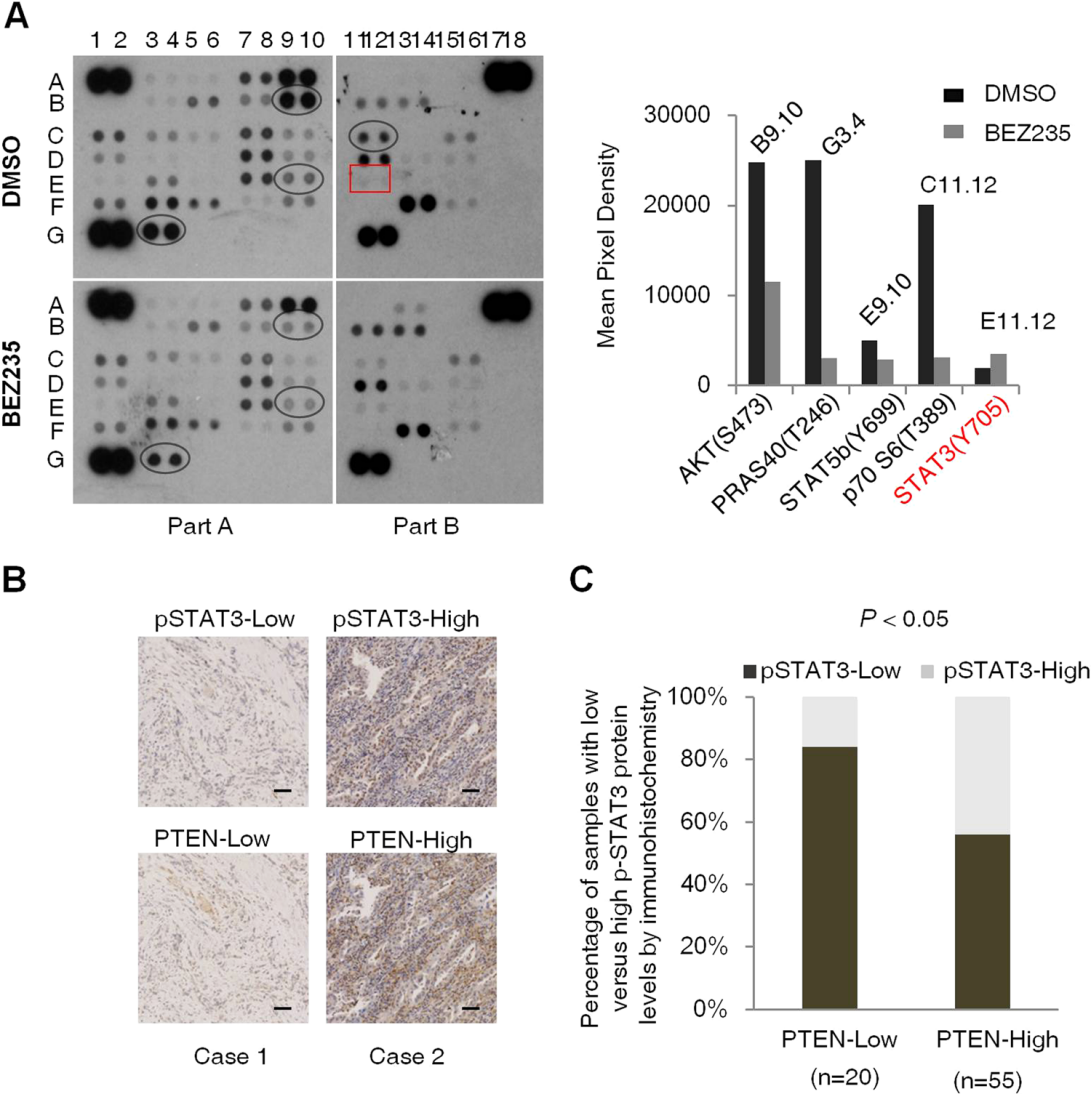
STAT3 activity is inhibited in PTEN-deficient cancer cells. **A** Phosphokinase antibody array blots using lysates of control and BEZ235-treated HGC-27 cells (left panel) and the changes in the expression levels of PI3K/AKT/mTOR signaling molecules and p-STAT3 activity before and after BEZ235 treatment (right panel). **B** Representative immunohistochemistry (IHC) images showing in situ PTEN and p-STAT3 expression in two patients with gastric cancer. Scale bars, 50 µm. **C** Correlation between PTEN and p-STAT3 expression in 75 gastric cancer patients. *P* < 0.05, calculated by Mann–Whitney tests.

Since the PI3K/AKT/mTOR signaling pathway is located at a crucial junction of multiple oncogenic signals, its inhibition activates several compensatory pathways that limit the therapeutic effects of targeted drugs. These compensatory signals in turn depend on the genetic background of the tumor cells. To verify this hypothesis, we analyzed the effect of PI3K/mTOR inhibition on signaling pathways in the PTEN-deficient gastric cancer HGC-27 cells using a phosphokinase array, which included antibodies against 43 phosphoproteins (Supplementary Table 1). As shown in Fig. 1A, the BEZ235-treated cells showed a significant inhibition of the PI3K/AKT/mTOR signaling molecules and an increase in p-STAT3^Tyr705^ levels, indicating that re-activation of the PI3K/AKT/mTOR pathway in PTEN-deficient cancer cells may involve inhibition of STAT3 activity. To prove this hypothesis, we analyzed the expression levels of PTEN and STAT3 kinase activity in tumor tissues from 75 gastric cancer patients. Twenty patients showed low PTEN expression, of which 16 (80%) also had low levels of p-STAT3^Tyr705^. In contrast, only 26 of the 55 patients (47%) with high PTEN expression showed low p-STAT3^Tyr705^ expression in the tumors (Fig. 1B, C). A positive correlation (*R*^2^ = 0.3076, Supplementary Fig. 1) between low PTEN and low p-STAT3^Tyr705^ expression indicated that PTEN deficiency suppresses STAT3 activity.

### PI3K/AKT/mTOR inhibition activates STAT3 in PTEN-deficient cancer cells

**Fig. 2:**
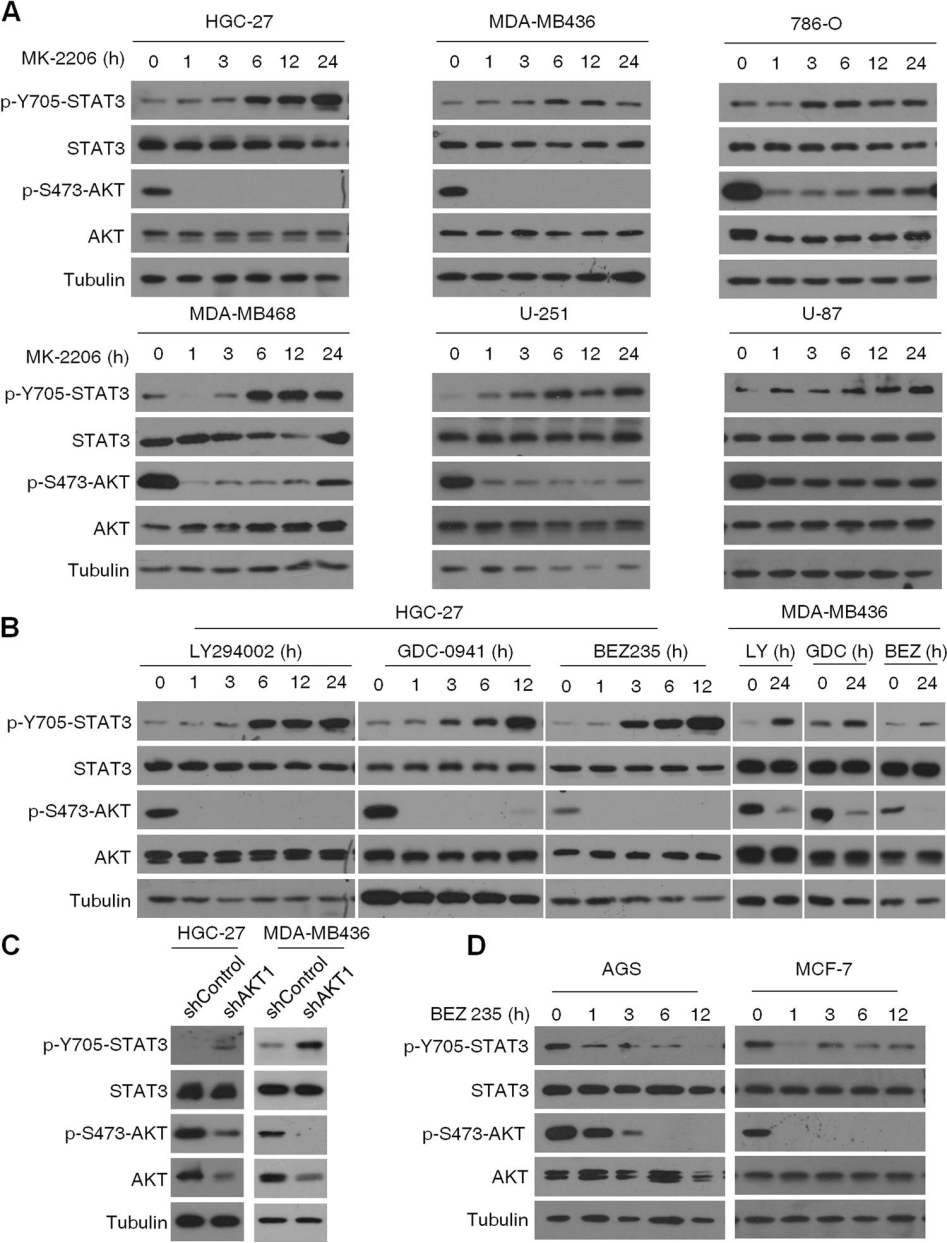
Inhibition of the PI3K/AKT/mTOR signaling pathway in PTEN-deficient cancer cells activates STAT3. **A** Immunoblot showing expression levels of p-STAT3, T-STAT3, p-AKT, and T-AKT in HGC-27, MDA-MB-436, 786-O, MDA-MB-468, U87, and U251 cells treated with 1 µM MK-2206 for varying durations. **B** Immunoblot showing expression levels of p-STAT3, T-STAT3, p-AKT, and T-AKT in HGC-27 (left panel) and MDA-MB-436 cells (right panel) treated with LY294002 (40 μM), GDC-0941 (1 μM), or BEZ235 (100 nM) for the indicated durations. **C** Immunoblot showing expression levels of p-STAT3, T-STAT3, p-AKT, and T-AKT in AKT1-knockdown HGC-27 and MDA-MB436 cells. **D** Immunoblot showing expression levels of p-STAT3, T-STAT3, p-AKT, and T-AKT in AGS and MCF-7 cells treated with BEZ235 (100 nM) for the indicated durations.

To determine whether PI3K/AKT/mTOR inhibition activates STAT3 in different PTEN-deficient cancer cells, we treated the gastric cancer cell line HGC-27, breast cancer cell lines MDA-MB468 and MDA-MB436, renal cell carcinoma cell line 786-O, and melanoma cell lines U-251 and U-87 with the AKT kinase inhibitor MK2206. Apart from inhibiting AKT kinase activity, MK2206 also increased STAT3 phosphorylation in a time-dependent manner (Fig. 2A). To rule out any specific effect of MK2206, we also treated HGC-27 and MDA-MB436 cells with the pan-PI3K inhibitor GDC0941, the PI3Kα/δ/β inhibitor LY294002, and the PI3K/mTOR kinase dual inhibitor BEZ235, and found that p-STAT3^Tyr705^ levels were increased with a corresponding decrease in AKT phosphorylation (Fig. 2B). To further prove that STAT3 activation was the result of PI3K/AKT/mTOR blockade rather than an off-target effect of the different inhibitors, we knocked down AKT1 expression in HGC-27 and MDA-MB-436 cells using shRNA interference, and detected increased STAT3 phosphorylation (Fig. 2C). In PTEN expression positive cell lines such as gastric cancer AGS and breast cancer MCF-7, the increased p-STAT3^Tyr705^ levels upon PI3K/mTOR inhibition was not detected (Fig. 2D). These results indicate that the PI3K/AKT/mTOR pathway blocks STAT3 activation in the PTEN-deficient cancer cells, and inhibition of this pathway induces STAT3 activity.

### Feedback activation of STAT3 limits the effect of PI3K/mTOR inhibitors on PTEN-deficient cancer cells

**Fig. 3:**
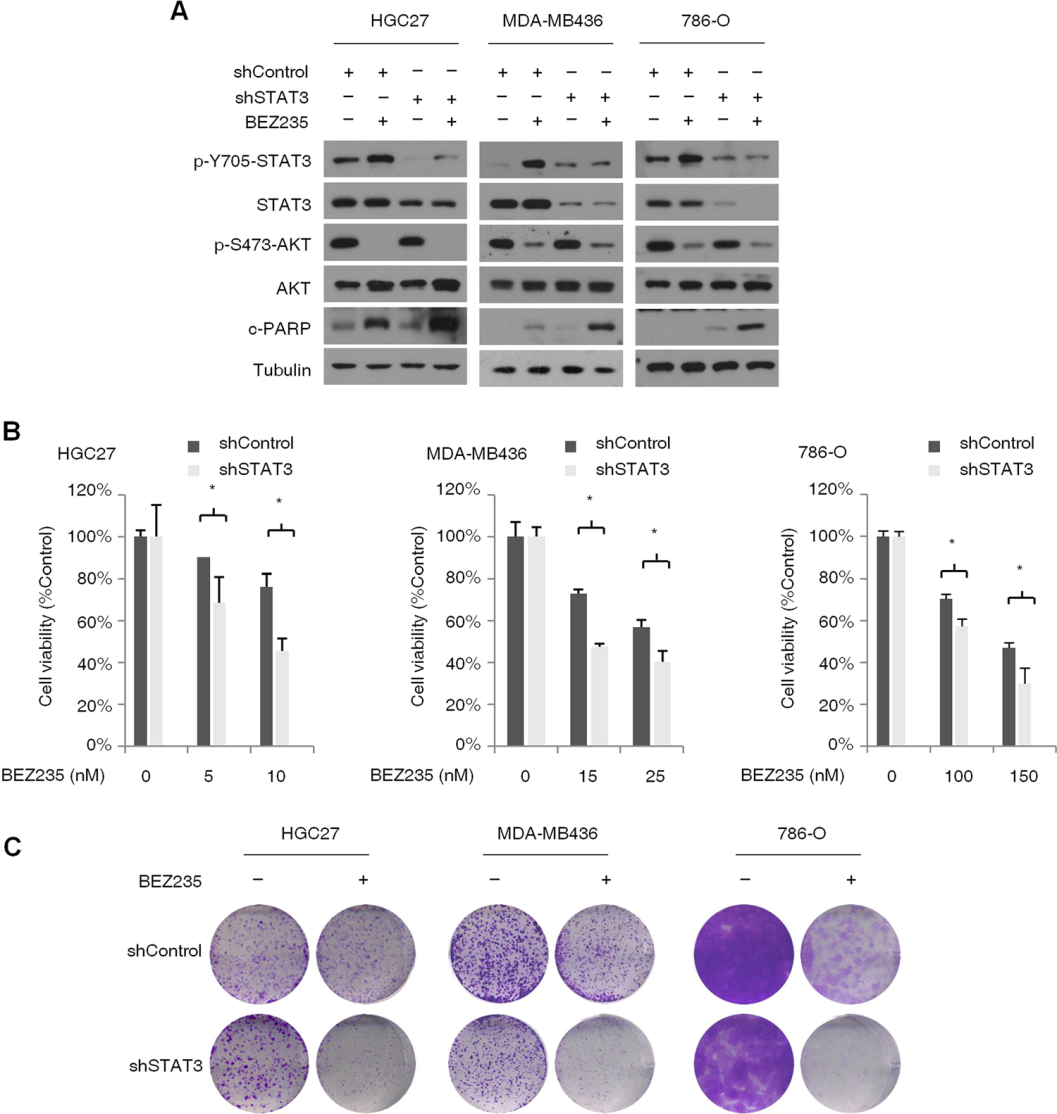
STAT3 activation attenuates the inhibitory effects of PI3K/mTOR inhibitor BEZ235 on PTEN-deficient cancer cells. **A** Immunoblot (left panel) showing expression levels of p-STAT3, T-STAT3, p-AKT, T-AKT, and cleaved-PARP (c-PARP) in the control and STAT3-knockdown HGC-27, MDA-MB-436, and 786-O cells treated with BEZ235 (100 nM) for 24 h. **B** Percentage of viable control and STAT3-knockdown HGC-27, MDA-MB-436, and 786-O cells after treatment with different concentrations of BEZ235 for 72 h. Error bars represent the means ± SD of triplicates. *P < 0.05. **C** Representative pictures showing crystal violet-stained colonies formed by the control and STAT3-knockdown HGC-27, MDA-MB-436, and 786-O cells treated with the indicated concentrations of BEZ235 for 14 days.

Studies show that feedback activation of STAT3 triggers resistance to EGFR, HER2, ALK, MEK, and histone deacetylase (HDAC) inhibitors. To determine whether STAT3 activation in PTEN-deficient cancer cells can also limit the effects of PI3K/AKT/mTOR inhibitors, we knocked down STAT3 in the HGC-27, MDA-MB436, and 786-O cells, and treated the wild-type and knockdown cells with BEZ235. While STAT3 knockdown alone had no significant effect on the expression levels of apoptosis markers cleaved poly (ADP ribose) polymerase (c-PARP), the latter were markedly upregulated in the BEZ235-treated STAT3 knockdown cells (Fig. 3A), indicating that STAT3 activation possibly limits the inhibitory effects of BEZ235 on cancer cell proliferation. Consistent with this hypothesis, STAT3 knockdown significantly augmented the BEZ235-induced decrease in the viability of cancer cells (Fig. 3B). In addition, the colony formation assay showed that STAT3 knockdown significantly enhanced the anti-clonogenic effects of BEZ235 treated for 14 days (Fig. 3C), indicating that STAT3 activation modulates resistance to long-term application of PI3K/mTOR inhibitors. These results show that feedback activation of STAT3 is involved in restraining the response to PI3K/AKT/mTOR inhibitors in the PTEN-deficient cancer cells.

### PI3K/mTOR inhibitor activates STAT3 through MIF/JAK1

**Fig. 4:**
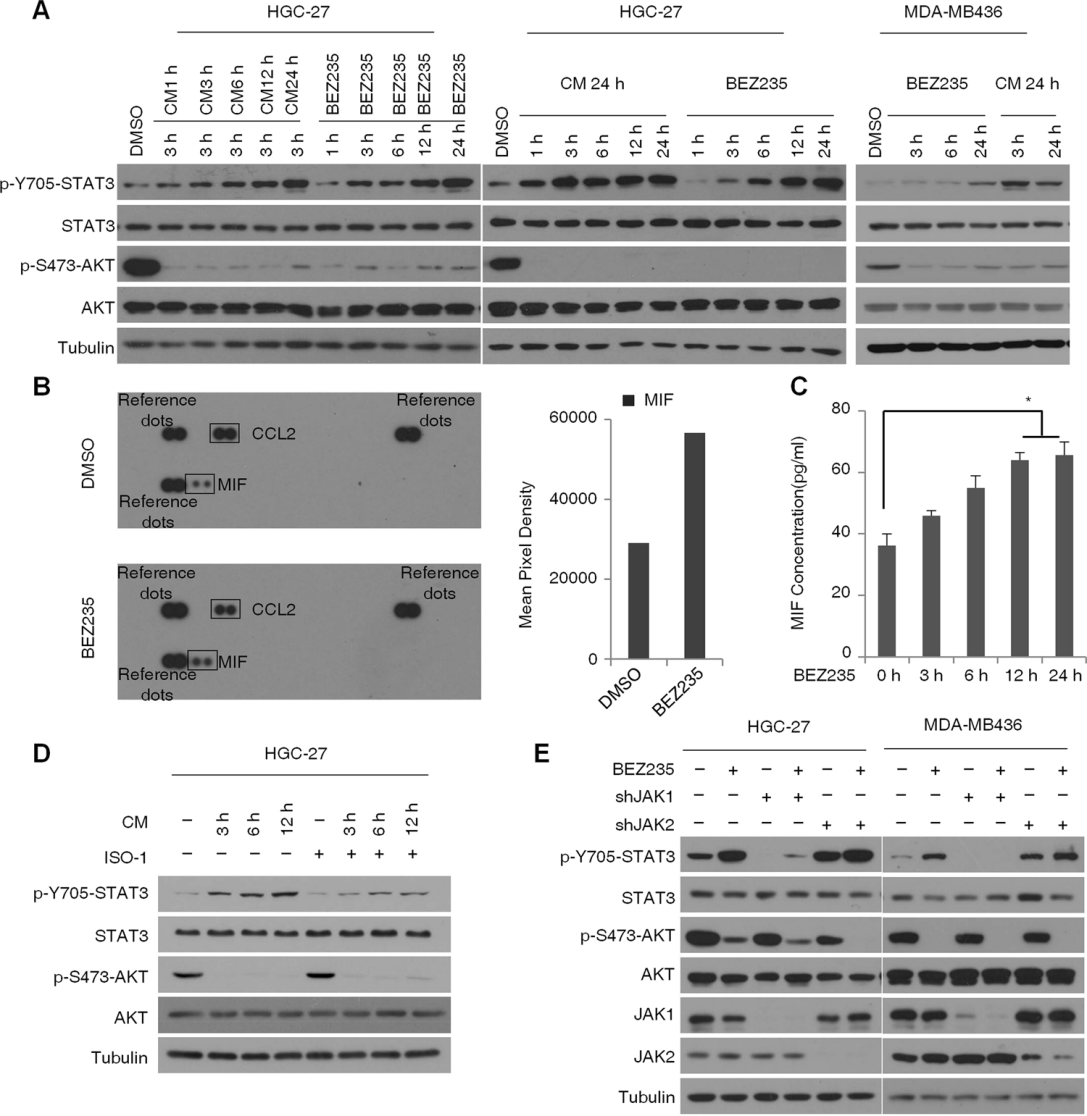
PI3K/mTOR inhibitor BEZ235 activates STAT3 through MIF and JAK1. **A** Immunoblots showing expression levels of p-STAT3, T-STAT3, p-AKT, and T-AKT in HGC27 cells cultured for 3 h in CM of BEZ235-treated (100 nM for indicated durations) cells (left panel), HGC27 cells (middle panel), and MDA-MB-436 cells (right panel) cultured for the indicated durations in 24 h CM of BEZ235-treated (100 nM) cells BEZ235-treated cells were used as controls. **B** Human cytokine array blot (left panel) showing the secreted cytokine profile of control and BEZ235-treated (100 nM for 24 h) HGC-27 cells. The bar graph shows fold change in secreted MIF levels. **C** Bar graphs showing the levels of MIF secretion in CM of HGC-27 cells treated with 100 nM BEZ235 for indicated durations. Levels of MIF secretion were measured by ELISA and are shown as means ± SD of triplicates. *P < 0.05. **D** Immunoblot showing expression levels of p-STAT3, T-STAT3, p-AKT, and T-AKT in HGC-27 cells pre-treated with ISO-1 (10 μM) for 2 h, and cultured in BEZ235-treated (100 nM) CM alone or in combination with ISO-1(10 μM) for indicated durations. **E** Immunoblot showing expression levels of p-STAT3, T-STAT3, p-AKT, and T-AKT in the control, JAK1 and JAK2-knockdown HGC-27 and MAD-MB436 cells treated with BEZ235 (100 nM) for 24 h.

Several cytokines and growth factors are known to activate STAT3. As shown in Fig. 1A, the drug-induced feedback activation of STAT3 was time dependent and peaked at 24 h, suggesting a temporal regulatory effect of cytokines. To examine this possibility, we cultured HGC-27 and MDA-MB436 cells with the conditioned media (CM) of BEZ235-treated respective cells collected at different time points. STAT3 activity was significantly elevated in HGC-27 cells cultured in CM collected at indicated durations. Similar STAT3 activity was detected in the cells cultured in the 24 h-CM for 3 h or treated with BEZ235 for 24 h, while STAT3 was not significantly activated in MDA-MB436 cells after 3 h BEZ235 treatment (Fig. 4A), indicating the presence of secreted factors that stimulated STAT3 activity. To identify these factors, we analyzed the cytokine profile of the CM and detected significantly high levels of the macrophage migration inhibitory factor (MIF) (Fig. 4B), which is known to stimulate STAT3 kinase activity. To gain further insight into the kinetics of MIF secretion into the CM of HGC-27 cells treated with BEZ235, an ELISA was performed to test the MIF protein level in the supernatant of cells. A significant increase in MIF secretion was found after 12 h of BEZ235 treatment (Fig. 4C). To determine whether elevation in MIF is the mechanism underlying BEZ235-induced STAT3 activation, we treated the HGC-27 cells with the MIF inhibitor ISO-1 at different time points, which partly inhibited the effects of BEZ235 (Fig. 4D). Therefore, we speculate that BEZ235 activates STAT3 to some degree by increasing MIF levels. To elucidate the role of JAK1 or JAK2 in STAT3 activation following PI3K/mTOR inhibition, we knocked down JAK1 and JAK2 in the HGC-27 and MDA-MB-436 cells. While JAK1 knockdown selectively blocked STAT3 activation in both cell lines, STAT3 was still activated in the BEZ235-treated JAK2 knockdown cells (Fig. 4E). These results indicate that JAK1, rather than JAK2 is involved in BEZ235-induced STAT3 activation in PTEN-deficient cancer cells.

### Co-targeting STAT3 and PI3K/mTOR reverses feedback activation of STAT3 and sensitizes cancer cells to BEZ235

**Fig. 5:**
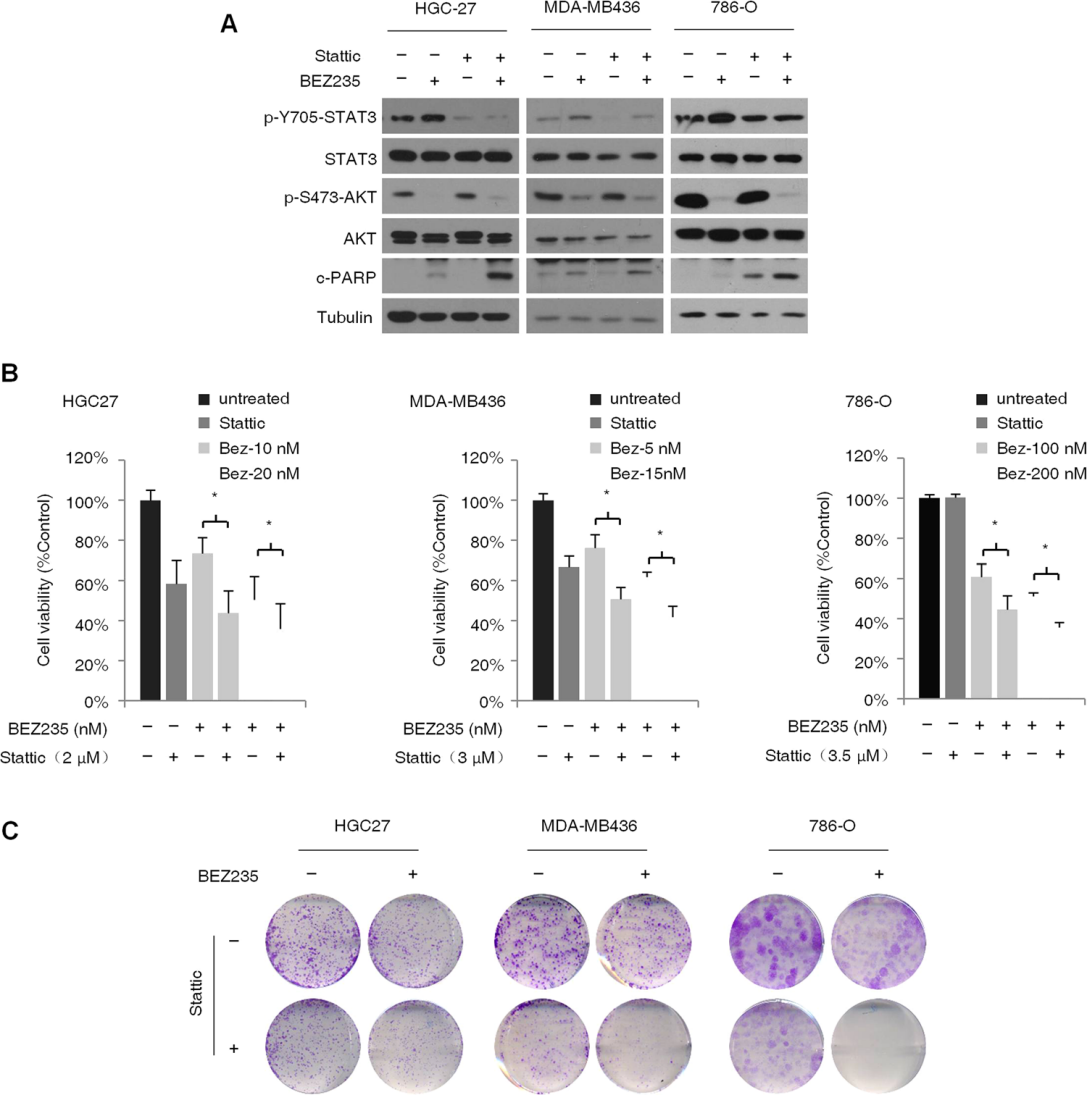
Simultaneous inhibition of PI3K/mTOR and STAT3 activity shows synergistic inhibitory effect on PTEN-deficient cancer cells. **A** Immunoblot showing expression levels of p-STAT3, T-STAT3, p-AKT, T-AKT, and c-PARP in HGC-27, MDA-MB-436, and 786-O cells treated with BEZ235 (100 nM) and/or Stattic (5 μM) for 24 h. **B** Percentage of viable HGC-27, MDA-MB-436, and 786-O cells treated with indicated concentration of BEZ235 and/or Stattic for 72 h. Error bars represent means ± SD from triplicates. *P < 0.05. **C** Representative pictures showing crystal violet-stained colonies formed by the HGC-27, MDA-MB-436, and 786-O cells treated with BEZ235 and/or Stattic for 14 days.

We next determined whether targeting STAT3 and the PI3K/mTOR pathway simultaneously blocked the feedback activation of STAT3 and sensitized PTEN-deficient cancer cells to BEZ235. To this end, HGC-27, MDA-MB436, and 786-O cells were treated with BEZ235 and/or the STAT3 inhibitor Stattic. While both drugs upregulated the expression of c-PARP when used alone, the combination treatment significantly enhanced apoptosis in all cell lines. In addition, Stattic effectively inhibited STAT3 in the presence of BEZ235 (Fig. 5A). These findings suggested that co-treatment with Stattic and BEZ235 reduced cancer cell resistance to BEZ235 due to STAT3 activation. Furthermore, both Stattic and BEZ235 inhibited proliferation of the PTEN-deficient cells individually, which underscores the oncogenic role of both STAT3 and PI3K/AKT/mTOR signaling pathways, and acted synergistically when used in combination (Fig. 5B). Consistent with this, Stattic significantly augmented the inhibitory effect of BEZ235 treatment for 14 days on the colony-forming abilities of all three PTEN-deficient cell lines (Fig. 5C). Taken together, the feedback activation of the STAT3 signaling pathway in PTEN-deficient cancer cells limits the therapeutic efficiency of PI3K/mTOR inhibitors, which can be circumvented by targeting the PI3K and STAT3 pathways simultaneously.

### Co-targeting STAT3 and PI3K/mTOR inhibits tumor xenograft growth in vivo

**Fig. 6:**
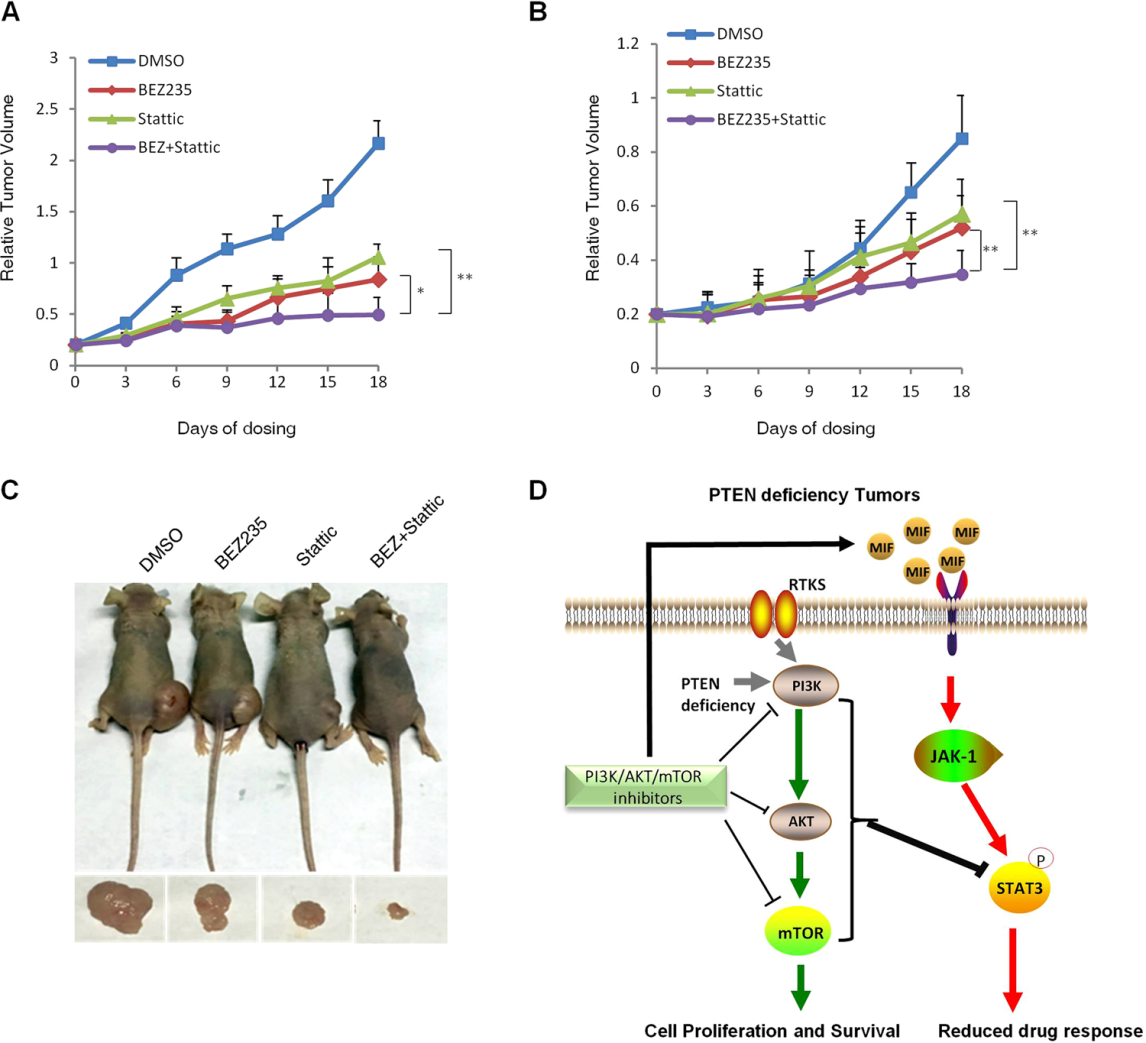
Combined inhibition of PI3K/mTOR and STAT3 activity significantly inhibits the growth of tumor xenografts. Growth curve of **A** HGC-27 and **B** MDA-MB-436 xenografts in mice treated with BEZ235 (30 mg/kg) and/or Stattic (3.75 mg/kg) for 20 days. The relative tumor volumes measured every 3 days are plotted. Error bars represent means ± SD (*n* = 6 mice per group). *P < 0.05. **C** Representative images of living nude mice injected with HGC-27 cells treated with BEZ235, or Stattic, or BEZ and Stattic or DMSO as indicated (upper panel). Stripped tumors are shown (lower panel). **D** Schematic representation of feedback activation of STAT3 limits the response to PI3K/AKT/mTOR inhibitors in PTEN-deficient cancer cells. Inhibition of the PI3K/AKT/mTOR signaling pathway led to the feedback activation of STAT3, which attenuated the anti-proliferative effects of PI3K/AKT/mTOR inhibitors in the PTEN-deficient cancer cells. PI3K/mTOR inhibitors increased the expression and secretion of macrophage migration inhibitory factor (MIF) and activated the JAK1/STAT3 signaling pathway. Both cancer cells and in vivo tumor xenografts showed that the combined inhibition of PI3K/AKT/mTOR and STAT3 activity enhanced the inhibitory effect of BEZ235 on the proliferation of PTEN-deficient cancer cells. The reactivated STAT3 limits the repression of PI3K/AKT/mTOR inhibitors on PTEN-deficient tumor growth.

In order to validate our in vitro findings and evaluate the in vivo therapeutic effects of PI3K/mTOR/STAT3 inhibition, we established xenograft models of HGC-27 and MDA-MB436 in nude mice. The mice were treated with BEZ235 and/or STAT3 daily for 20 days. Both drugs inhibited the growth of the HGC-27 xenografts compared to that in untreated controls mice, and the combination treatment was more effective compared to BEZ235 alone (Fig. 6A, C). Furthermore, the body weight of the mice did not significantly decrease, indicating that the combination treatment was tolerable to the animals (data not shown). Similar results were also achieved in the MDA-MB436 tumor xenograft model (Fig. 6B). Taken together, simultaneous targeting of STAT3 and the PI3K/mTOR pathway can sensitize PTEN-deficient cancer cells to BEZ235, and inhibit tumor growth in vivo (Fig. 6D).

## Discussion

In this study, we found that PI3K/AKT/mTOR inhibitors upregulated MIF expression and secretion in several PTEN-deficient cancer cells, and activated STAT3 activity through JAK1, eventually limiting the effects of these inhibitors. Contradictory to studies showing that AKT inhibitors can target multiple RTKs in various cancer cell types, the PI3K/mTOR inhibitor BEZ235 did not significantly alter the activity of different STAT3-regulating RTKs in PTEN-deficient gastric cancer cells. A previous study showed that dual inhibition of PI3K/mTOR in BEZ235-resistant breast cancer cells triggered a positive feedback response and activated the JAK2/STAT5 pathway, resulting in increased IL-8 secretion and drug resistance^32^. Although we did not detect high levels of IL-6 and IL-8 in the conditioned medium (CM) of BEZ235-treated HGC-27 cells, the cells later cultured in this medium showed high STAT3 activity, indicating that other factors were involved in STAT3 activation. Cytokine array analysis showed a marked elevation in the pleiotropic cytokine MIF, and blocking the latter with ISO-1 significantly inhibited STAT3 activation by BEZ235. Furthermore, knockdown of JAK1 rather than JAK2 completely inhibited BEZ235-induced STAT3 activation, indicating that JAK1 is the primary mediator. Taken together, BEZ235 activates STAT3 via the MIF/JAK1 pathway. MIF is a pleiotropic cytokine that regulates inflammation, cell proliferation, and differentiation, and also promotes tumor cell proliferation, angiogenesis, and metastasis by activating the STAT3, ERK, and PI3K/AKT signaling pathways^33–35^. Interestingly, a MEK inhibitor induced drug resistance in KRAS-mutant colorectal cancer cells by upregulating the MIF/STAT3 axis^36^. In our study however, MIF inhibition did not completely block STAT3 activity, indicating the involvement of other regulatory cytokines.

Phosphorylated STAT3 molecules enter the nucleus and bind to their specific DNA effector elements in the anti-apoptotic genes B-cell lymphoma 2 (Bcl-2) and B-cell lymphoma-extra large (Bcl-xL), and therefore inhibit apoptosis^37,38^. Consistent with this, knocking down STAT3 in the BEZ235-treated cancer cells significantly augmented the pro-apoptotic effect of BEZ235. Therefore, STAT3 activation can lower the efficacy of BEZ235 by inhibiting apoptosis, which was confirmed by cell proliferation and colony formation assays as well. The feedback activation of STAT3 by PI3K/mTOR inhibitors raises the possibility that simultaneously targeting PI3K/mTOR and STAT3 activity in PTEN-deficient cancer cells can synergistically inhibit tumor progression. Therefore, we blocked the feedback activation of STAT3 kinase using the specific inhibitor Stattic, which enhanced the anti-proliferative effects of BEZ235 in the PTEN-deficient cells. Despite these encouraging preliminary results, there are still certain limitations as far as clinical application is concerned. For instance, although some small molecule inhibitors of STAT3 are in the preclinical and early clinical phases of testing, they are still at a nascent stage compared to other TKIs, and their clinical effects are therefore largely unknown^39–41^. In view of this and the upstream role of JAK1 in the feedback activation of STAT3, the combination of PI3K/AKT/mTOR inhibitors and pan-JAK1/JAK2 inhibitors such as INCB018424^42,43^ should also be tested in PTEN-deficient cancer cells.

In conclusion, targeted cancer therapies have different drug resistance mechanisms based on the genetic background of the tumors. The PI3K/AKT/mTOR signaling pathway is an effective therapeutic target in PTEN-deficient cancer cells, but its inhibitors can induce resistance via feedback activation of STAT3, which provides an empirical basis for combining both PI3K/AKT/mTOR and STAT3 inhibitors.

## Materials and methods

### Cell lines and cell culture

The gastric cancer cell line HGC-27 was obtained from the Cell Resource Center, Peking Union Medical College. Breast cancer cell lines MDA-MB436 and MDA-MB468, renal cell carcinoma cell line 786-O, and melanoma cell lines U-251 and U-87 were obtained from the American Type Culture Collection (ATCC). All cell lines were authenticated by short tandem repeat (STR) analysis and confirmed to be free of mycoplasma within the past year. All cell lines were grown in Dulbecco’s modified Eagle’s medium (DMEM) supplemented with 10% (vol/vol) FBS and penicillin/streptomycin (penicillin, 100 IU/ml; streptomycin, 100 μg/ml) at 37 °C under 5% CO_2_. The cell lines were mycoplasma-free and authenticated by analyzing their morphology and growth profile.

### Antibodies and reagents

Antibodies against STAT3 (no. 12640), phospho-STAT3-Tyr705 (no. 9145), AKT (no. 9272), phospho-Akt-Ser473 (no. 4060), JAK1 (no. 3344), JAK2 (no. 3230), PARP (no. 9532), and PTEN (no. 9188) were purchased from Cell Signaling Technology. The anti-γ-Tubulin antibody (sc-7396) was purchased from Santa Cruz biotechnology. MK2206, GDC0941, LY294002, BEZ235, and Stattic were from Selleck Chemicals.

### Lentiviral transduction

HEK293T cells were transfected with 6 µg DNA (3 µg pLKO.1-shRNA, 2.25 µg psPAX2, and 0.75 µg pMD2.G) using 40 µl Lipofectamine 3000 in 300 µl Opti-MEM media (Invitrogen, USA). The culture medium was replaced with fresh medium after 16 h, and the supernatant containing viral particles were collected 24 and 48 h after transfection. After centrifuging at 3000 rpm for 10 min, the cleared supernatants were filtered through a 0.45-µm filter, and stored at −80 °C. The HGC-27 or MDA-MB-436 cells were infected with the virus using polybrene (6 μg/ml), and the transduced cells were screened 48 h later using puromycin (3 µg/ml). The clones with stable knockdown of STAT3, AKT1, JAK1, or JAK2 were identified and verified by western blotting. The shRNA were synthesized by Biomed (Beijing, China). Detailed information on the shRNA sequences may be found in Supplementary Table S2.

### Phosphokinase antibody array

HGC-27 cells were seeded in a 100-mm dish at the density of 1.5 × 10^6^ per dish, and treated with DMSO or BEZ235 for 24 h. The cells were washed with cold PBS and lysed in NP40 buffer, and 300 µg lysates were spotted on the human phospho-kinase antibody array (#ARY003B, R&D Systems) membranes. After incubating overnight with the antibody cocktail according to manufacturer’s instructions, the membranes were washed with the suitable buffer, developed using a chemiluminescent reagent and exposed to X-ray film.

### Western blotting

The suitably treated cells were homogenized in NP-40 lysis buffer containing a protease inhibitor cocktail (Roche), and protein concentration in the lysates were determined by BCA assay (Pierce Chemical, Rockford, IL, USA). Equal amount of proteins per sample were subjected to SDS–PAGE and transferred to PVDF membranes (Immobilon-P, Millipore). The membranes were blocked with 5% nonfat milk for 1 h at room temperature, and incubated overnight with primary antibodies at 4 °C, followed by HRP-conjugated anti-rabbit/mouse/goat IgG for 1 h at room temperature. The positive bands were detected using enhanced chemiluminescence (ECL) detection reagent.

### Secreted factor array and ELISA

CM was prepared from the control and BEZ235-treated HGC-27 or MDA-MB436 cells. Briefly, the cells were seeded in 100-mm culture plates at the density of 1.5 × 10^6^ per dish in complete medium. The following day, the medium was discarded and replaced with serum-free media. After overnight serum starvation, the cells were treated with DMSO or 100 nM BEZ235 for varying durations. The CM was collected at each time point and centrifuged for 5 min at 3000 rpm to remove cellular debris, and aliquots were stored at −80 °C until use. The CM was analyzed using the Proteome Profiler Human Cytokine Array containing antibodies against 36 human cytokines (#ARY005B, R&D Systems) according to the manufacturer’s instructions. MIF secretion in CM treated with BEZ235 at the indicated time point was measured by ELISA using DuoSet ELISA kit (R&D Systems, Minneapolis, MN, USA) according to the manufacturer’s instructions.

### Cell viability assay

Cells were seeded in 96-well plates at the density of 1500 cells per well in complete DMEM, and cultured overnight. After treating the cells with DMSO or the indicated inhibitors for 72 h, their viability was determined using the Cell Counting Kit-8 assay kit (CCK-8, Dojindo Laboratories, Tokyo, Japan) according to the manufacturer’s instructions. The absorbance of individual wells was determined at 450 nm, and the OD value of the treatment groups were normalized to that of untreated controls. Three independent experiments were performed, and each condition was tested in triplicate.

### Colony formation assay

Cells were plated in six-well plates at the density of 500–1000 cells per well, and treated with DMSO or the indicated inhibitors for 14 days. The ensuing colonies were washed with ice-cold PBS and stained with 0.5% crystal violet in 25% methanol. Images were acquired with a digital camera.

### Patient samples and immunohistochemistry

The patient samples were collected after informed consent in accordance with the Declaration of Helsinki, and the research protocol was reviewed and approved by the Ethics Committee of Beijing Institute of Biotechnology. A human gastric cancer tissue microarray (Cat^#^ HStm-Ade150CS-01) comprising of 75 paired tumor and normal gastric mucosa tissue samples was purchased from Outdo Biotech, Shanghai, China (Supplementary Table S3). The samples were deparaffinized, rehydrated, and treated with 3% hydrogen peroxide for 20 min to quench endogenous peroxidase activity. Following antigen retrieval by microwaving, the samples were blocked with 10% normal serum and incubated overnight with anti-PTEN (1:50; Cell Signaling) and anti-p-STAT3 (1:100; Cell Signaling) antibodies at 4 °C. After incubating with the biotinylated anti-rabbit secondary antibody and streptavidin–horseradish peroxidase, the staining was developed using 3,3′-diaminobenzidine and counterstained with hematoxylin. The widely accepted German semiquantitative scoring system in considering the staining intensity and area extent was used. Each specimen was assigned a score according to the intensity of the nucleic, cytoplasmic, and/or membrane staining (no-staining = 0; weak staining = 1, moderate staining = 2, strong staining = 3) and the extent of stained cells (0% = 0, 1–24% = 1, 25–49% = 2, 50–74% = 3, 75–100% = 4). The final immunoreactive score was determined by multiplying the intensity score with the extent of score of stained cells, ranging from 0 to 12. The 75 samples were classified into two groups (low PTEN scores from 0 to 4, 20 samples; high PTEN scores from 5 to 12, 55 samples).

### Animal studies

All animal experiments were performed in accordance with the protocols approved by the Institutional Animal Care and Use Committee at Beijing Institute of Biotechnology. To establish the xenografts, 5 × 10^6^ HGC-27 or MDA-MB436 cells were injected subcutaneously in 6-week-old female BALB/c nude mice (Vital River Laboratory, Beijing, China) in serum-free medium. Once the tumors grew to 100–150 mm^3^, the tumor-bearing mice were randomized into the control, BEZ235 (30 mg/kg orally), Stattic (3.75 mg/kg intra-peritoneally), and BEZ235 + Stattic combination groups, and given the requisite drug doses daily for the indicated duration. The tumors were measured every 3 days using calipers, and the volume was calculated as (length × width^2^)/2.

### Statistical analysis

The data were represented as the mean ± SD of at least three independent experiments, and compared using a two-tailed unpaired Student’s *t*-test, or Mann–Whitney test. *P*-value < 0.05 was considered statistically significant.

## Supplementary information

Supplemental Material

## References

[1] Haber DA, Gray NS, Baselga J (2011). The evolving war on cancer. Cell.

[2] Sellers WR (2011). A blueprint for advancing genetics-based cancer therapy. Cell.

[3] Flaherty KT (2010). Inhibition of mutated, activated BRAF in metastatic melanoma. N. Engl. J. Med..

[4] O’Brien SG (2003). Imatinib compared with interferon and low-dose cytarabine for newly diagnosed chronic-phase chronic myeloid leukemia. N. Engl. J. Med..

[5] Daub H, Specht K, Ullrich A (2004). Strategies to overcome resistance to targeted protein kinase inhibitors. Nat. Rev. Drug Discov..

[6] Engelman JA (2007). MET amplification leads to gefitinib resistance in lung cancer by activating ERBB3 signaling. Science (New York, NY).

[7] Sos ML (2009). PTEN loss contributes to erlotinib resistance in EGFR-mutant lung cancer by activation of Akt and EGFR. Cancer Res..

[8] Sheppard K, Kinross KM, Solomon B, Pearson RB, Phillips WA (2012). Targeting PI3 kinase/AKT/mTOR signaling in cancer. Crit. Rev. Oncog..

[9] Miller BW (2015). FDA approval: idelalisib monotherapy for the treatment of patients with follicular lymphoma and small lymphocytic lymphoma. Clin. Cancer Res..

[10] Yardley DA (2013). Everolimus plus exemestane in postmenopausal patients with HR(+) breast cancer: BOLERO-2 final progression-free survival analysis. Adv. Ther..

[11] Li J (1997). PTEN, a putative protein tyrosine phosphatase gene mutated in human brain, breast, and prostate cancer. Science (New York, NY).

[12] Maehama T, Dixon JE (1999). PTEN: a tumour suppressor that functions as a phospholipid phosphatase. Trends Cell Biol..

[13] Guertin DA (2009). mTOR complex 2 is required for the development of prostate cancer induced by Pten loss in mice. Cancer Cell.

[14] Podsypanina K (2001). An inhibitor of mTOR reduces neoplasia and normalizes p70/S6 kinase activity in Pten+/- mice. Proc. Natl Acad. Sci. USA.

[15] Jia S (2008). Essential roles of PI(3)K-p110beta in cell growth, metabolism and tumorigenesis. Nature.

[16] Ni J (2012). Functional characterization of an isoform-selective inhibitor of PI3K-p110beta as a potential anticancer agent. Cancer Discov..

[17] Wee S (2008). PTEN-deficient cancers depend on PIK3CB. Proc. Natl Acad. Sci. USA.

[18] Schwartz S (2015). Feedback suppression of PI3Kalpha signaling in PTEN-mutated tumors is relieved by selective inhibition of PI3Kbeta. Cancer Cell.

[19] Brachmann SM (2009). Specific apoptosis induction by the dual PI3K/mTor inhibitor NVP-BEZ235 in HER2 amplified and PIK3CA mutant breast cancer cells. Proc. Natl Acad. Sci. USA.

[20] O’Brien C (2010). Predictive biomarkers of sensitivity to the phosphatidylinositol 3’ kinase inhibitor GDC-0941 in breast cancer preclinical models. Clin. Cancer Res..

[21] Serra V (2008). NVP-BEZ235, a dual PI3K/mTOR inhibitor, prevents PI3K signaling and inhibits the growth of cancer cells with activating PI3K mutations. Cancer Res..

[22] Weigelt B, Warne PH, Downward J (2011). PIK3CA mutation, but not PTEN loss of function, determines the sensitivity of breast cancer cells to mTOR inhibitory drugs. Oncogene.

[23] Li J (2013). The AKT inhibitor AZD5363 is selectively active in PI3KCA mutant gastric cancer, and sensitizes a patient-derived gastric cancer xenograft model with PTEN loss to Taxotere. J. Transl. Med..

[24] Carver BS (2011). Reciprocal feedback regulation of PI3K and androgen receptor signaling in PTEN-deficient prostate cancer. Cancer Cell.

[25] Mulholland DJ (2011). Cell autonomous role of PTEN in regulating castration-resistant prostate cancer growth. Cancer Cell.

[26] Bowman T, Garcia R, Turkson J, Jove R (2000). STATs in oncogenesis. Oncogene.

[27] Bromberg JF (1999). Stat3 as an oncogene. Cell.

[28] Yu H, Pardoll D, Jove R (2009). STATs in cancer inflammation and immunity: a leading role for STAT3. Nat. Rev. Cancer.

[29] Zhao C (2016). Feedback Activation of STAT3 as a Cancer Drug-Resistance Mechanism. Trends Pharmacol. Sci..

[30] Lee HJ (2014). Drug resistance via feedback activation of Stat3 in oncogene-addicted cancer cells. Cancer Cell.

[31] Zeng H (2016). Feedback activation of leukemia inhibitory factor receptor limits response to histone deacetylase inhibitors in breast cancer. Cancer Cell.

[32] Britschgi A (2012). JAK2/STAT5 inhibition circumvents resistance to PI3K/mTOR blockade: a rationale for cotargeting these pathways in metastatic breast cancer. Cancer Cell.

[33] Lue H, Kleemann R, Calandra T, Roger T, Bernhagen J (2002). Macrophage migration inhibitory factor (MIF): mechanisms of action and role in disease. Microbes Infect..

[34] Lv W (2016). Macrophage migration inhibitory factor promotes breast cancer metastasis via activation of HMGB1/TLR4/NF kappa B axis. Cancer Lett..

[35] Ohta S (2012). Macrophage migration inhibitory factor (MIF) promotes cell survival and proliferation of neural stem/progenitor cells. J. Cell Sci..

[36] Cheon SK (2018). Macrophage migration inhibitory factor promotes resistance to MEK blockade in KRAS mutant colorectal cancer cells. Mol. Oncol..

[37] Alas S, Bonavida B (2003). Inhibition of constitutive STAT3 activity sensitizes resistant non-Hodgkin’s lymphoma and multiple myeloma to chemotherapeutic drug-mediated apoptosis. Clin. Cancer Res..

[38] Real PJ (2002). Resistance to chemotherapy via Stat3-dependent overexpression of Bcl-2 in metastatic breast cancer cells. Oncogene.

[39] Beebe JD, Liu JY, Zhang JT (2018). Two decades of research in discovery of anticancer drugs targeting STAT3, how close are we?. Pharmacol. Ther..

[40] Johnson DE, O’Keefe RA, Grandis JR (2018). Targeting the IL-6/JAK/STAT3 signalling axis in cancer. Nat. Rev. Clin. Oncol..

[41] Wong ALA (2017). Do STAT3 inhibitors have potential in the future for cancer therapy?. Expert Opin. Investig. Drugs.

[42] Mascarenhas J, Hoffman R (2012). Ruxolitinib: the first FDA approved therapy for the treatment of myelofibrosis. Clin. Cancer Res..

[43] Verstovsek S (2012). A double-blind, placebo-controlled trial of ruxolitinib for myelofibrosis. N. Engl. J. Med..

