# Supplementary material for: Feedback activation of STAT3 limits the response to PI3K/AKT/mTOR inhibitors in PTEN-deficient cancer cells
Source: Oncogenesis. 2021 Jan 5;10(1):8. doi: 10.1038/s41389-020-00292-w (PMC7801611; doi:10.1038/s41389-020-00292-w)
Supplement: Supplementary file 1 — Supplemental Material [file 41389_2020_292_MOESM1_ESM.docx]

Table S1, Related to Figure 1. A complete list of kinases induction folds included in Human Phospho-Kinase Array treated with BEZ235 for 24 hr

| Membrane/Coordinate | Target/Control | Phosphorylation Site | Fold induction  with BEZ235 |
| --- | --- | --- | --- |
| A-A1, A2 | Reference Spot |  | 1 |
| A-A3, A4 | p38α | T180/Y182 | 0.74 |
| A-A5, A6 | ERK1/2 | T202/Y204, T185/Y187 | 0.74 |
| A-A7, A8 | JNK 1/2/3 | T183/Y185, T221/Y223 | 0.91 |
| A-A9, A10 | GSK-3α/β | S21/S9 | 0.57 |
| B-A13, A14 | p53 | S392 | 2.71 |
| B-A17, A18 | Reference Spot |  |  |
| A-B3, B4 | EGFR | Y1086 | 0.75 |
| A-B5, B6 | MSK1/2 | S376/S360 | 0.89 |
| A-B7, B8 | AMPKα1 | T183 | 0.59 |
| A-B9, B10 | Akt 1/2/3 | S473 | 0.19 |
| B-B11, B12 | Akt 1/2/3 | T308 | 1.13 |
| B-B13, B14 | p53 | S46 | 1,70 |
| A-C1, C2 | TOR | S2448 | 0.93 |
| A-C3, C4 | CREB | S13 | 0.84 |
| A-C5, C6 | HSP27 | S78/S82 | 0.84 |
| A-C7, C8 | AMPKα2 | T172 | 0.83 |
| A-C9, C10 | β-Catenin |  | 0.85 |
| B-C11, C12 | p70 S6 Kinase | T389 | 0.37 |
| B-C13, C14 | p53 | S15 | 0.98 |
| B-C15, C16 | c-Jun | S63 | 1.01 |
| A-D1, D2 | Src | Y419 | 0.98 |
| A-D3, D4 | Lyn | Y397 | 1 |
| A-D5, D6 | Lck | Y394 | 1 |
| A-D7, D8 | STAT2 | Y689 | 0.55 |
| A-D9, D10 | STAT5a | Y694 | 0.89 |
| B-D11, D12 | p70 S6 Kinase | T421/S424 | 1.02 |
| B-D13, D14 | RSK1/2/3 | S380/S386/S377 | 1.09 |
| B-D15, D16 | eNOS | S1177 | 0.82 |
| A-E1, E2 | Fyn | Y420 | 1 |
| A-E3, E4 | Yes | Y426 | 1.15 |
| A-E5, E6 | Fgr | Y412 | 1 |
| A-E7, E8 | STAT6 | Y641 | 0.95 |
| A-E9, E10 | STAT5b | Y699 | 0.59 |
| B-E11, E12 | STAT3 | Y705 | 1.81 |
| B-E13, E14 | p27 | T198 | 1 |
| B-E15, E16 | PLC-γ1 | Y783 | 1 |
| A-F1, F2 | Hck | Y411 | 0.89 |
| A-F3, F4 | Chk-2 | T68 | 0.99 |
| A-F5, F6 | FAK | Y397 | 0.98 |
| Membrane/Coordinate | Target/Control | Phosphorylation Site |  |
| A-F7, F8 | PDGFRβ | Y751 | 1 |
| A-F9, F10 | STAT5a/b | Y694/Y699 | 0.89 |
| B-F11, F12 | STAT3 | S727 | 1 |
| B-F13, F14 | WNK1 | T60 | 0.58 |
| B-F15, F16 | PYK2 | Y402 | 0.84 |
| A-G1, G2 | Reference Spot |  |  |
| A-G3, G4 | PRAS40 | T246 | 0.58 |
| A-G9, G10 | PBS(Negative Control) |  |  |
| B-G11, G12 | HSP60 |  | 1 |
| B-G17, G18 | PBS(Negative Control) |  |  |

Table S2. shRNA sequences used in this study

| Primer name | Primer sequence from 5’ to 3’ |
| --- | --- |
| shRNA-STAT3-F | CCGGCGGCGTCCAGTTCACTACTAACTGCAGTTAGTAGTGAACTGGACGCCGTTTTTG |
| shRNA-STAT3-R | AATTCAAAAACGGCGTCCAGTTCACTACTAACTGCAGTTAGTAGTGAACTGGACGCCG |
| shRNA-AKT1-F | CCGGGGACTACCTGCACTCGGAGAACTCGAGTTCTCCGAGTGCAGGTAGTCCTTTTTG |
| shRNA-AKT1-R | AATTCAAAAAGGACTACCTGCACTCGGAGAACTCGAGTTCTCCGAGTGCAGGTAGTCC |
| shRNA-JAK1-F | CCGGCGTTCTCTACTACGAAGTGATCTCGAGATCACTTCGTAGTAGAGAACGTTTTTG |
| shRNA-JAK1-R | AATTCAAAAACGTTCTCTACTACGAAGTGATCTCGAGATCACTTCGTAGTAGAGAACG |
| shRNA-JAK2-F | CCGGGCAACTTGGCAAGGGTAATTTCTCGAGAAATTACCCTTGCCAAGTTGCTTTTTG |
| shRNA-JAK2-R | AATTCAAAAAGCAACTTGGCAAGGGTAATTTCTCGAGAAATTACCCTTGCCAAGTTGC |

Table S3, Related to Figure 1. Gastric Adenocarcinoma tissue microarray characteristics

| Patient ID | Age | Gender | Pathological Grade | PTEN Score | p-STAT3^Tyr705^ Score |
| --- | --- | --- | --- | --- | --- |
| A1 | 67 | M | III | 4 | 4 |
| A3 | 63 | M | II-III | 8 | 4 |
| A5 | 43 | M | II | 8 | 12 |
| A7 | 69 | M | II-III | 8 | 0 |
| A9 | 57 | M | I-II | 8 | 8 |
| A11 | 50 | F | II-III | 8 | 4 |
| A13 | 74 | M | II | 8 | 8 |
| A15 | 53 | M | II-III | 12 | 4 |
| B1 | 53 | M | II | 4 | 4 |
| B3 | 69 | M | II | 12 | 12 |
| B5 | 54 | M | II-III | 8 | 0 |
| B7 | 63 | F | III | 4 | 4 |
| B9 | 67 | M | II | 12 | 4 |
| B11 | 77 | M | II | 12 | 9 |
| B13 | 76 | M | II-III | 2 | 0 |
| B15 | 77 | M | II | 8 | 0 |
| C1 | 47 | M | I-II | 4 | 3 |
| C3 | 69 | M | II | 6 | 2 |
| C5 | 75 | M | II | 8 | 2 |
| C7 | 72 | F | II | 6 | 8 |
| C9 | 47 | F | II-III | 8 | 6 |
| C11 | 76 | F | III | 12 | 4 |
| C13 | 63 | F | III | 12 | 8 |
| C15 | 73 | F | III | 0 | 8 |
| D1 | 51 | M | III | 2 | 8 |
| D3 | 78 | F | II | 6 | 8 |
| D5 | 61 | M | II-III | 12 | 8 |
| D7 | 51 | F | III | 12 | 0 |
| D9 | 75 | M | III | 4 | 0 |
| D11 | 55 | M | II | 8 | 2 |
| D13 | 76 | F | II-III | 4 | 4 |
| D15 | 60 | M | II | 12 | 6 |
| E1 | 56 | M | II | 0 | 4 |
| E3 | 61 | M | II | 8 | 8 |
| E5 | 55 | M | II-III | 12 | 8 |
| E7 | 78 | M | III | 12 | 4 |
| E9 | 50 | F | II | 12 | 4 |
| E11 | 46 | M | I-III | 8 | 8 |
| E13 | 67 | M | II | 12 | 6 |
| E15 | 60 | M | II-III | 12 | 0 |
| F1 | 64 | F | II-III | 8 | 8 |
| F3 | 50 | M | II-III | 12 | 9 |
| F5 | 63 | M | II-III | 6 | 8 |
| F7 | 47 | F | III | 4 | 3 |
| F9 | 55 | M | III | 12 | 4 |
| F11 | 55 | M | II-III | 6 | 8 |
| F13 | 57 | M | II-IV | 8 | 4 |
| F15 | 46 | M | III | 8 | 4 |
| G1 | 59 | F | II-III | 8 | 8 |
| G3 | 64 | F | II-III | 8 | 8 |
| G5 | 56 | F | II-III | 8 | 8 |
| G7 | 69 | F | II-III | 12 | 4 |
| G9 | 62 | M | II-III | 8 | 2 |
| G11 | 68 | M | I-III | 3 | 12 |
| G13 | 50 | M | II-III | 8 | 12 |
| G15 | 67 | M | II-III | 12 | 4 |
| H1 | 87 | M | III | 0 | 12 |
| H3 | 56 | M | III | 4 | 2 |
| H5 | 69 | M | II-III | 4 | 0 |
| H7 | 71 | M | III | 4 | 4 |
| H9 | 80 | M | II-III | 8 | 0 |
| H11 | 72 | M | III | 4 | 4 |
| H13 | 48 | M | III | 12 | 8 |
| H15 | 65 | M | III | 12 | 8 |
| I1 | 51 | M | III | 4 | 3 |
| I3 | 69 | M | II-III | 4 | 4 |
| I5 | 40 | F | III | 12 | 4 |
| I7 | 75 | F | III | 12 | 8 |
| I9 | 61 | M | II | 12 | 12 |
| I11 | 50 | M | II-III | 8 | 4 |
| I13 | 70 | M | III | 12 | 0 |
| I15 | 58 | M | II | 12 | 12 |
| J1 | 52 | M | III | 4 | 0 |
| J3 | 54 | M | II-III | 8 | 4 |
| J5 | 80 | F | II-III | 12 | 12 |

**Supplementary Figure 1:** Regression analysis comparing p-STAT3 with PTEN expression (low PTEN scores from 0-4) in gastric cancer tissues. *n*=20.
